# Real-World Outcome Analysis of Patients With Stage IV NSCLC Treated With Tyrosine Kinase and Immune Checkpoint Inhibitors

**DOI:** 10.1016/j.jtocrr.2023.100524

**Published:** 2023-05-04

**Authors:** Ryo Ariyasu, Sho Kakuto, Keiki Miyadera, Takahiro Akita, Ayu Kiritani, Ryosuke Tsugitomi, Yoshiaki Amino, Ken Uchibori, Satoru Kitazono, Noriko Yanagitani, Makoto Nishio

**Affiliations:** Department of Thoracic Medical Oncology, The Cancer Institute Hospital, Japanese Foundation for Cancer Research, Tokyo, Japan

**Keywords:** Non–small cell lung cancer, Long-term survival data, Real-world, A tyrosine kinase inhibitor, Immune checkpoint inhibitor

## Abstract

**Introduction:**

Only a few reports have determined whether recently advanced anticancer drugs, particularly next-generation tyrosine kinase inhibitors (TKIs) and immune checkpoint inhibitors (ICIs), prolong the survival of patients with NSCLC in the real world.

**Methods:**

To evaluate the association between recently advanced drugs and patient survival, survival data of 2078 patients with stage IV NSCLC from 1995 to 2022 were analyzed in the present study. The patients were classified into the following six groups in terms of the date of diagnosis: period A, 1995 to 1999; period B, 2000 to 2004; period C, 2005 to 2009; period D, 2010 to 2014; period E, 2015 to 2019; and period F, 2000 to 2022. They were further grouped in terms of *EGFR* mutation and *ALK* fusion.

**Results:**

The median overall survival (mOS) times were 8.9, 11.0, 13.6, 17.9, and 25.2 months in periods A to E, respectively, and the mOS time was not reached in period F. This time was significantly longer in period E than in period D (25.2 versus 17.9 mo, *p* < 0.005). Moreover, the mOS times of patients with *EGFR* mutation, those with *ALK* fusion, and those without both alterations were significantly longer in period E than in period D (46.0 versus 32.0 mo, *p* < 0.005; not reached versus 36.2 mo, *p* = 0.018; 14.6 versus 11.7 mo, *p* < 0.005). The history of treatment with next-generation TKIs and ICIs was found to be associated with overall survival.

**Conclusions:**

The survival of patients with NSCLC was improved from period D to period E, regardless of the presence of driver gene alteration. We found that next-generation TKIs and ICIs may be associated with improvements in overall survival.

## Introduction

NSCLC is one of the leading causes of cancer-related deaths worldwide and in Japan.^1^ Recently, there have been advances in anticancer drugs for NSCLC, including cytotoxic chemotherapy,^2^ anti-vascular endothelial growth factor therapy,^3^ molecular target agents,^4^^,^^5^ and immune checkpoint inhibitors (ICIs).^6^^,^^7^ Moreover, the introduction of a new class of anticancer drugs, tyrosine kinase inhibitors (TKIs), has been explored to prolong the survival of patients with NSCLC.^8^ In addition, in our previous study, we reported an improvement in the survival of patients with stage IV NSCLC from 1995 to 2017 at our institution.^9^ Notably, the survival was particularly improved in patients diagnosed in 2010 to 2014, and this might be attributed to the use of TKIs—a class of anticancer drugs.

After 2014, next-generation TKIs and ICIs were approved for NSCLC and became available in Japan. For example, the second-generation ALK TKI alectinib was approved in 2014,^10^ the anti–programmed cell death protein 1 (PD-1) antibody nivolumab was approved in 2015,^6^^,^^7^ and the third-generation EGFR TKI osimertinib was approved in 2016.^11^ Furthermore, the introduction of next-generation TKIs and anti–PD-1/programmed death-ligand 1 (PD-L1) antibodies (a new class of anticancer drugs) was expected to prolong the survival of patients with NSCLC in clinical trials. Nevertheless, it is still unclear whether these drugs prolong the survival of patients with NSCLC in clinical practice. Some reports have indicated the advantages of the use of ICIs; however, the evidence remains insufficient.^12^ In addition, in our previous study, we could not analyze the improvement in survival with the use of these drugs because of the short follow-up time.^9^

In the present study, we aimed to update the survival data of our previous study by incorporating 5-year follow-up data. Furthermore, we aimed to analyze the survival data in terms of the driver gene alterations to assess the effects of next-generation TKIs and anti–PD-1/PD-L1 antibodies on patient survival. Owing to the increasing importance of treatment strategies and prognosis on the basis of driver gene alterations in patients with NSCLC in recent years, such an analysis is crucial for understanding the disease.^13^ By updating survival data grouped by driver gene alterations with sufficient follow-up time, we were able to more accurately assess the contribution of next-generation TKIs and anti–PD-1/PD-L1 antibodies to the survival of patients with NSCLC.

In the present study, we updated the survival data of patients with stage IV NSCLC from 1995 to 2022 at the Cancer Institute Hospital of the Japanese Foundation for Cancer Research. Moreover, we analyzed the survival data of patients with stage IV NSCLC, particularly those grouped by driver gene alterations, and the association between their survival and treatment history.

## Materials and Methods

### Study Design and Patient Population

The primary objective of this retrospective analysis study was to analyze whether next-generation TKIs and ICIs prolonged the survival of patients with NSCLC. We collected data of patients with stage IV NSCLC (based on the seventh edition of the TNM classification for lung cancer) at the Department of Thoracic Oncology, Cancer Institute Hospital of Japanese Foundation for Cancer Research from our clinical database. The study included patients diagnosed with having NSCLC from April 1995 to March 2022 and those receiving treatments that included best supportive care. The following data of the patients were collected: age, sex, smoking history, Eastern Cooperative Oncology Group performance status (PS), pathologic diagnosis, driver mutation, date of diagnosis, date of death, and treatment history. Notably, overall survival (OS) was calculated as the duration from the date of diagnosis to the date of death, and surviving patients were excluded in April 2022. Furthermore, patients lost to follow-up were excluded on the date of the last follow-up. The history of treatment with drugs approved in April 2022 in Japan was identified, and the drugs were as follows: EGFR TKIs, gefitinib, erlotinib, afatinib, dacomitinib, and osimertinib; ALK TKIs, crizotinib, alectinib, ceritinib, brigatinib, and lorlatinib; angiogenesis inhibitors, bevacizumab, and ramucirumab; ICIs, nivolumab, pembrolizumab, atezolizumab, and durvalumab; other molecular target drugs, crizotinib and entrectinib for *ROS1*, dabrafenib and trametinib for *BRAF*, tepotinib and capmatinib for *MET*, and selpercatinib for *RET*.

The patients were classified into the following six groups in terms of the date of diagnosis: period A, 1995 to 1999; period B, 2000 to 2004; period C, 2005 to 2009; period D, 2010 to 2014; period E, 2015 to 2019; and period F, 2020 to 2022. They were further grouped in terms of the driver gene alterations: those with EGFR mutation, those with ALK fusion, and those without both alterations. Furthermore, OS was estimated for each group. The institutional review board of our hospital approved this study (institutional review board number 2022-GB-042). Informed consent was waived because of the retrospective nature of the study, and an opt-out option was included.

### Statistical Analysis

Patient characteristics were compared using Fisher’s exact test. OS was estimated using the Kaplan–Meier method, and survival curves were compared using the log-rank test. Propensity score matching was used to adjust for the following known patient characteristics at baseline between each period: age, sex, smoking history, PS, and pathologic diagnosis. The propensity score was calculated using multiple logistic regression analysis. Moreover, Cox regression analysis was used to evaluate the association between patient characteristics and OS in univariate analysis. In addition, variables with *p* values of less than 0.05 in the univariate analysis were included in the multivariate analysis. SPSS Statistics for Windows version 24 (IBM Corp., Armonk, NY) was used for all statistical analyses.

## Results

### Patient Characteristics

Overall, 2080 patients with stage IV NSCLC were treated at our hospital from 1995 to 2022. Two patients refused to have their medical records reused. At the cutoff date, 289 patients were alive, and 149 patients were lost to follow-up. The patient characteristics are described in Table 1. The percentage of older patients increased from periods C to D and from periods D to E (*p* < 0.005 and *p* < 0.005, respectively), the incidence of adenocarcinoma increased from periods C to D (*p* < 0.005), and the number of patients with PS equals 0 or 1 increased from periods B to C (*p* < 0.005). No considerable differences were found in the percentage of female patients and never smokers among the evaluated periods. The rates of *EGFR* mutation and *ALK* fusion were almost same in periods D, E, and F (29%–32% and 6%–9%, respectively). Notably, two patients had both *EGFR* mutation and *ALK* fusion. The rates of PD-L1 expression of greater than or equal to 50% on the basis of the 22C3 assay were 13% and 23% in periods E and F, respectively (Table 1).

**Table 1:** Patient Characteristics

Characteristics	Period A 1995-99	Period B 2000-04	Period C 2005-09	Period D 2010-14	Period E 2015-19	Period F 2020-22
N	149	237	378	544	561	209
Age						
<75 (%)	135 (91)	218 (92)	356 (94)	482 (89)	448 (80)	173 (83)
≥75 (%)	14 (9)	19 (8)	22 (6)	62 (11)	113 (20)	36 (17)
Sex						
Female (%)	50 (34)	85 (36)	145 (38)	214 (39)	214 (38)	76 (36)
Male (%)	99 (66)	152 (64)	233 (62)	330 (61)	347 (62)	133 (64)
Smoking status						
Never (%)	26 (17)	57 (24)	102 (27)	178 (33)	184 (33)	60 (29)
Current, former or unknown (%)	123 (83)	180 (76)	276 (73)	366 (67)	377 (67)	149 (71)
Histology						
Adenocarcinoma (%)	110 (74)	186 (78)	278 (74)	445 (82)	449 (80)	165 (79)
Others (%)	39 (26)	51 (22)	100 (26)	99 (18)	112 (20)	44 (21)
ECOG PS						
0 or 1 (%)	106 (71)	172 (73)	326 (86)	459 (84)	452 (81)	166 (79)
≥2 or unknown (%)	43 (29)	65 (27)	52 (14)	85 (16)	109 (19)	43 (21)
EGFR mutation						
Positive (%)			49 (13)	174 (32)	173 (31)	61 (29)
ALK fusion						
Positive (%)			2 (1)	47 (9)	31 (6)	12 (6)
PD-L1 expression						
Negative (%)					57 (10)	46 (22)
1%–49% (%)					35 (6)	38 (18)
50% or higher (%)					71 (13)	48 (23)
Unknown (%)					398 (71)	77 (37)

ECOG PS, Eastern Cooperative Oncology Group performance status; PD-L1, programmed death-ligand 1.

### OS of All Patients With Stage IV NSCLC

**Figure 1:**
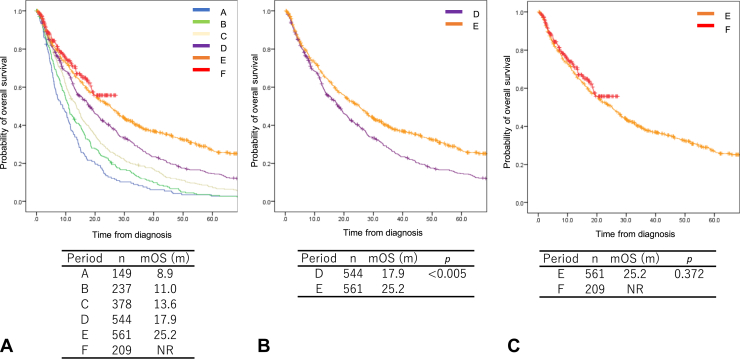
The overall survival of all patients with stage IV NSCLC. (*A*) Periods A–F; (*B*) periods D and E; (*C*) periods E and F. mOS, median overall survival; NR, not reached.

The OS of each period is described in Figure 1. The median OS (mOS) was 8.9, 11.0, 13.6, 17.9, and 25.2 months in periods A to E, respectively, and it was not reached (NR) in period F (Fig. 1*A*). Given that our previous report mainly analyzed patients in periods A to C, the analysis in the present study was performed for patients in periods D to F using 5-year updated data. Notably, the mOS time was significantly longer in period E than in period D (25.2 versus 17.9 mo, *p* < 0.005; Fig. 1*B*). In contrast, no significant difference was found between periods E and F in terms of the mOS time (25.2 mo versus NR, *p* = 0.372; Fig. 1*C*).

### OS With Propensity Score Matching

**Figure 2:**
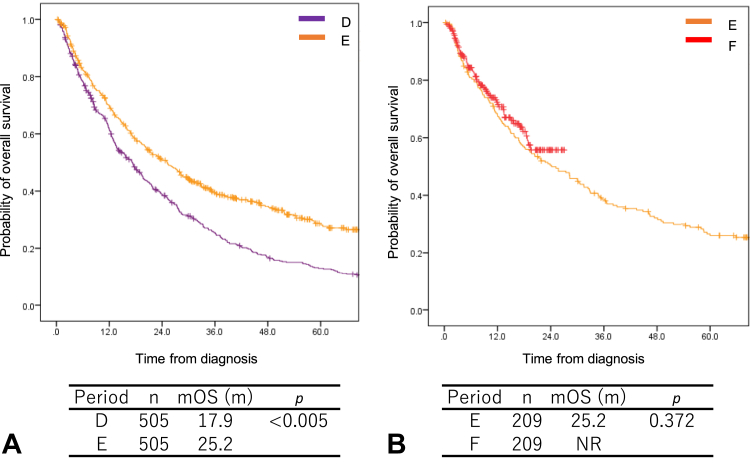
The overall survival of all patients with stage IV NSCLC with propensity score matching. (*A*) Periods D and E; (*B*) periods E and F. mOS, median overall survival; NR, not reached.

The patients were paired by propensity score matching between periods D and E and between periods E and F. The matching variables were age, sex, smoking history, PS, and histology. After propensity score matching, the survival data of 505 patients were compared between periods D and E and those of 209 patients were compared between periods E and F. We found that the mOS time was significantly longer in period E than in period D (25.2 versus 17.9 mo, *p* < 0.005; Fig. 2*A*). No significant difference was found between periods E and F in terms of the mOS time (25.2 mo versus NR, *p* = 0.372; Fig. 2*B*).

### OS Grouped by EGFR Mutation and ALK Fusion

**Figure 3:**
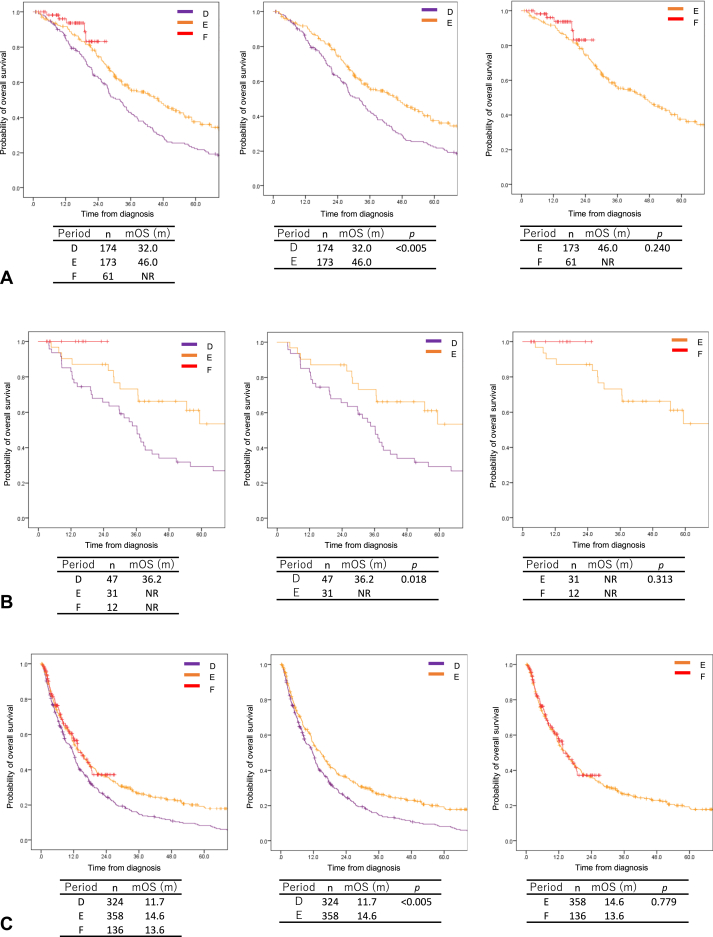
The overall survival in patients grouped by *EGFR* mutation and *ALK* fusion. (*A*) Patients with *EGFR* mutation; (*B*) patients with *ALK* fusion; (*C*) patients without both alterations. mOS, median overall survival; NR, not reached.

In this study, the mOS times of patients grouped by *EGFR* mutation and *ALK* fusion were compared between each period. In periods D to F, the mOS times were 32.0 months, 46.0 months, and NR in the *EGFR*-positive group (Fig. 3*A*); 36.2 months, NR, and NR in the *ALK*-positive group (Fig. 3*B*); and 11.7, 14.6, and 13.6 months in the group lacking both alterations (Fig. 3*C*), respectively. Furthermore, the mOS times of patients with *EGFR* mutation, those with *ALK* fusion, and those without both alterations were significantly longer in period E than in period D (46.0 versus 32.0 mo, *p* < 0.005; NR versus 36.2 mo, *p* = 0.018; 14.6 versus 11.7 mo, *p* < 0.005, respectively). The mOS times were not significantly longer in period F than in period E in all groups (NR versus 46.0 mo, *p* = 0.240 in the *EGFR*-positive group; NR versus NR, *p* = 0.313 in *ALK* fusion; 13.6 versus 14.6 mo, *p* = 0.779 in the absence of both alterations; Fig. 3).

### Treatment History Grouped by EGFR Mutation and ALK Fusion

The treatment history of patients grouped by *EGFR* mutation and *ALK* fusion is found in Table 2. In the *EGFR* mutation-positive group, the percentage of patients receiving third-generation EGFR TKIs increased from 19.0% in period D to 60.1% in period E. In the *ALK* fusion-positive group, the percentage of patients receiving second- or third-generation ALK TKIs increased from 48.9% in period D to 96.8% in period E. In the absence of both alterations, the percentage of patients receiving ICIs increased from 8.3% in period D to 67.0% in period E. Notably, the percentage of patients receiving ICIs as first-line therapy increased from 0% in period D to 52.1% in period E. Moreover, the percentage of patients receiving other molecular target drugs increased from 0.6% in period D to 3.4% in period E (Table 2).

**Table 2:** Treatment History Grouped by *EGFR* Mutation and *ALK* Fusion

EGFR Mutation	Period D 2010-14	Period E 2015-19	Period F 2020-22
N	174	173	61
Any systemic treatment	174 (100)	169 (97.7)	61 (100)
EGFR-TKI	174 (100)	161 (93.1)	56 (91.8)
Third generation EGFR-TKI	33 (19.0)	104 (60.1)	53 (86.9)
Angiogenesis inhibitor	47 (27.0)	38 (22.0)	10 (16.4)
ICI	21 (12.1)	41 (23.7)	9 (14.8)

ALK fusion	Period D 2010-14	Period E 2015-19	Period F 2020-22
N	47	31	12
Any systemic treatment	47 (100)	31 (100)	12 (100)
ALK-TKI	41 (87.2)	30 (96.8)	12 (100)
Second or third generation ALK-TKI	23 (48.9)	30 (96.8)	12 (100)
Angiogenesis inhibitor	15 (31.9)	2 (6.5)	0
ICI	2 (4.3)	5 (16.1)	0

Absence of both alteration	Period D 2010-14	Period E 2015-19	Period F 2020-22
N	324	358	136
Any systemic treatment	261 (80.6)	293 (81.8)	121 (89.0)
Other molecular target drugs	2 (0.6)	12 (3.4)	11 (8.1)
Angio genesis inhibitor	61 (18.8)	57 (15.9)	11 (8.1)
ICI	27 (8.3)	240 (67.0)	111 (81.6)
First line monotherapy		44	19
First line chemo/immunotherapy		75	42
First line combination immunotherapy		6	44
Second or later line	27	115	6

ICI, immune checkpoint inhibitor; TKI, tyrosine kinase inhibitor.

### Clinical Factors Associated With OS

We analyzed the clinical factors associated with OS in each patient population grouped by *EGFR* mutation and *ALK* fusion. To precisely analyze the effects of ICIs, patients treated with other molecular target drugs were excluded from the analysis. The results of the univariate analysis indicated that PS and third-generation EGFR TKI therapy were associated with OS in the *EGFR* mutation-positive group. In addition, age, PS, histology, and second- and third-generation ALK TKI therapies were associated with OS in the *ALK* fusion group. Finally, age, PS, histology, any systemic therapy, angiogenesis inhibitor therapy, and ICI therapy were associated with OS in patients without both alterations. The results of the multivariate analysis indicated that PS and third-generation EGFR TKI therapy were associated with OS in the *EGFR* mutation group. In addition, PS, histology, and second- or third-generation ALK TKI therapy were associated with OS in the *ALK* fusion group. Finally, PS, histology, any systemic therapy, angiogenesis inhibitor therapy, and ICI therapy were associated with OS in patients without both alterations. The use of third-generation EGFR TKIs affected OS (hazard ratio [HR] = 0.38, 95% confidence interval [CI]: 0.29–0.50, *p* < 0.005) in the *EGFR* mutation group. Moreover, the use of second- or third-generation ALK TKIs affected OS (HR = 0.23, 95% CI: 0.12–0.44, *p* < 0.005) in the ALK fusion group. Notably, the use of any systemic treatment and ICI therapy had the greatest effect on OS (HR = 0.36, 95% CI: 0.27–0.47, *p* = 0.012; HR = 0.45, 95% CI: 0.37–0.54, *p* < 0.005) in patients without any alterations (Table 3).

**Table 3:** Clinical Factors Associated With Overall Survival in Patients Grouped by *EGFR* Mutation and *ALK* Fusion

(A) Univariate Analysis								
EGFR Mutation			ALK Fusion			Absence of Both Alteration		
Factor	HR	*p* Value	Factor	HR	*p* Value	Factor	HR	*p* Value
Age			Age			Age		
≥75	1.23 (0.86–1.77)	0.25	≥75	2.95 (1.05–8.27)	**0.04**	≥75	1.43 (1.16–1.78)	**<0.005**
<75			<75			<75		
Sex			Sex			Sex		
Male	1.09 (0.84–1.41)	0.51	Male	1.49 (0.83–2.67)	0.18	Male	1.10 (0.91–1.32)	0.35
Female			Female			Female		
Smoking history			Smoking history			Smoking history		
Past or unknown	0.93 (0.72–1.20)	0.58	Past or unknown	0.91 (0.51–1.63)	0.76	Past or unknown	1.17 (0.95–1.46)	0.14
Never			Never			Never		
PS = 2			PS = 2			PS = 2		
2 or unknown	1.93 (1.61–2.33)	**<0.005**	2 or unknown	1.60 (1.14–2.23)	**0.006**	2 or unknown	1.86 (1.69–2.05)	**<0.005**
0 or 1			0 or 1			0 or 1		
Pathology			Pathology			Pathology		
Adenocarcinoma	0.65 (0.30–1.37)	0.25	Adenocarcinoma	0.03 (0.01–0.18)	**<0.005**	Adenocarcinoma	0.76 (0.64–0.91)	**<0.005**
Others			Others			Others		
Any systemic treatment			Any systemic treatment			Any systemic treatment		
Yes	2.19 (0.31–15.6)	0.44	Yes	NA	NA	Yes	0.16 (0.13–0.19)	**<0.005**
No			No			No		
EGFR-TKI			ALK-TKI					
Yes	0.86 (0.41–1.83)	0.70	Yes	0.47 (0.20–1.10)	0.08			
No			No					
Third G EGFR-TKI			Second or third G ALK-TKI					
Yes	0.42 (0.32–0.55)	**<0.005**	Yes	0.23 (0.13–0.42)	**<0.005**			
No			No					
Angiogenesis inhibitor			Angiogenesis inhibitor			Angiogenesis inhibitor		
Yes	0.89 (0.67–1.17)	0.39	Yes	1.19 (0.62–2.30)	0.60	Yes	0.58 (0.46–0.72)	**<0.005**
No			No			No		
ICI			ICI			ICI		
Yes	0.76 (0.55–1.05)	0.09	Yes	0.97 (0.38–2.48)	0.96	Yes	0.37 (0.31–0.43)	**<0.005**
No			No			No		

(B) Multivariate Analysis								
EGFR Mutation			ALK Fusion			Absence of Both Alteration		
Factor	HR	*p* Value	Factor	HR	*p* Value	Factor	HR	*p* Value
			Age			Age		
			≥75	1.75 (0.58–5.29)	0.32	≥75	1.05 (0.84–1.31)	0.68
			<75			<75		
PS = 2			PS = 2			PS = 2		
2 or unknown	2.16 (1.79–2.62)	**<0.005**	2 or unknown	1.89 (1.34–2.67)	**<0.005**	2 or unknown	1.41 (1.26–1.59)	**<0.005**
0 or 1			0 or 1			0 or 1		
			Pathology			Pathology		
			Adenocarcinoma	0.05 (0.01–0.29)	**<0.005**	Adenocarcinoma	0.77 (0.64–0.93)	**0.005**
			Others			Others		
						Any systemic treatment		
						Yes	0.36 (0.27–0.47)	**<0.005**
						No		
Third G EGFR-TKI			Second or third G ALK-TKI					
Yes	0.38 (0.29–0.50)	**<0.005**	Yes	0.23 (0.12–0.44)	**<0.005**			
No			No					
						Angiogenesis inhibitor		
						Yes	0.73 (0.57–0.92)	**0.009**
						No		
						ICI		
						Yes	0.45 (0.37–0.54)	**<0.005**
						No		

*Note:* Differences were considered statistically significant at a probability value of less than 0.05 and are indicated in bold.

G, generation; HR, hazard ratio; ICI, immune checkpoint inhibitor; NA, not available; PS, performance score; TKI, tyrosine kinase inhibitor.

## Discussion

In this report, we analyzed the OS of patients with stage IV NSCLC from 1995 to 2022 using 5-year updated data. We found that survival was more improved in period E (2015–2019) compared with period D (2010–2014). Moreover, we found that the survival of patients with stage IV NSCLC continued to prolong after our previous report.^9^ Because our previous study analyzed patients in periods A to C, in this study, we analyzed patients in periods D to F. We also evaluated whether next-generation TKIs and ICIs prolonged the survival of patients with NSCLC in the real world.

Survival was found to be improved in each patient population grouped by *EGFR* mutation and *ALK* fusion from period D to period E. The Cox regression model revealed that the history of treatment with third-generation EGFR TKIs and second- or third-generation ALK TKIs and ICIs is associated with OS in each patient group. Moreover, the percentage of patients receiving these drugs increased from periods D to E. These data suggest that the introduction of such drugs improves the survival of patients with NSCLC.

Many studies have reported improvements in the survival of patients with NSCLC owing to the development of anticancer drugs. For example, Howlader et al.^8^ reported that the population-level mortality caused by NSCLC decreased in the United States. Moreover, other reports indicated that the use of new drug classes may improve real-world survival of such patients.^14^, ^15^, ^16^ These results are consistent with those of our study; however, they do not completely indicate the effects of third-generation EGFR TKIs and second- or third-generation ALK TKIs. We found that the association between next-generation TKIs and improvement in survival of patients with NSCLC would more precisely reflect the recent advances in their treatments. Some studies have reported improvements in survival from pre- to post-ICI periods, and this finding is consistent with that of the present study.^12^^,^^17^^,^^18^ Nevertheless, the previous studies did not analyze survival data in terms of *EGFR* mutation and *ALK* fusion. It is known that anti–PD-1/PD-L1 antibodies are not fully effective on NSCLC with *EGFR* mutation or *ALK* fusion^19^ and are often used for *EGFR* and *ALK* wild-type mutations. Accordingly, our analysis indicated that the grouping of survival data by *EGFR* mutation and *ALK* fusion would be more consistent with real-world clinical practice.

Many clinical trials have reported improvement in OS with the use of next-generation TKIs and anti–PD-1/PD-L1 antibodies.^11^^,^^20^, ^21^, ^22^ Nevertheless, clinical trials are known to have strict inclusion and exclusion criteria; therefore, their results cannot be applied to all patients in clinical practice. Hence, it is important to report improvements in the survival of such patients with real-world data. The results of the present study suggest that improvement in survival of patients with NSCLC with the introduction of next-generation TKIs and anti–PD-1/PD-L1 antibodies will encourage both practitioners and patients.

This study has some limitations. Owing to the single-center retrospective nature of the study, there may be some inherent biases, including patient selection bias. Nevertheless, analyzing data from a single institution enables consistency in data collection and treatment strategy. In addition, we used propensity score matching and multivariate analysis to minimize biases. Nevertheless, some unknown factors related to patient background and prognosis could not be analyzed. In our study cohort, the percentages of patients with PS of 0 or 1 and those receiving any systemic treatment were higher than those in another report.^23^ Our institution is a specialized cancer hospital, and many patients are referred to our institution after being diagnosed with having NSCLC at other hospitals. Therefore, notably, patients having poor PS and multiple comorbidities are relatively rare at our hospitals.

In the present study, we could not report the improvement in survival from period E (2015–2019) to period F (2020–2022). After 2020, novel molecular target drugs (such as selpercatinib and tepotinib) for rare driver gene alterations and the anti–CTLA-4 antibody ipilimumab were approved in Japan.^24^, ^25^, ^26^, ^27^ Nevertheless, we could not comprehensively analyze the effects of these drugs because of the small number of patients using the drugs and short follow-up time. Moreover, owing to the coronavirus disease 2019 pandemic in period F, the diagnosis and treatment of patients with NSCLC might have been delayed.^28^ In the present study, the total number of patients and the percentage of those with PS of 0 or 1 decreased from period E to F, which might have affected the survival analysis. In the future, we aim to prolong the follow-up time and clarify the effects of recent advancements in NSCLC drugs.

In conclusions, there was an improvement in the survival of patients with stage IV NSCLC from period D (2010–2014) to period E (2015–2019) on the basis of the 5-year updated data. Notably, this improvement was found in patients grouped by *EGFR* mutation and *ALK* fusion. We found that next-generation TKIs and anti–PD-1/PD-L1 antibodies may be associated with improvements in OS.

## CRediT Authorship Contribution Statement

**Ryo Ariyasu:** Conceptualization, Data curation, Formal analysis, Methodology, Resources, Writing—original draft.

**Sho Kakuto, Keiki Miyadera, Takahiro Akita, Ayu Kiritani, Ryosuke Tsugitomi, Yoshiaki Amino, Ken Uchibori, Satoru Kitazono, Noriko Yanagitani:** Resources, Writing—review and editing.

**Makoto Nishio:** Conceptualization, Methodology, Resources, Supervision, Writing—review and editing.
